# Ups and Downs of Viagra: Revisiting Ototoxicity in the Mouse Model

**DOI:** 10.1371/journal.pone.0079226

**Published:** 2013-11-14

**Authors:** Adrian Au, John Gerka Stuyt, Daniel Chen, Kumar Alagramam

**Affiliations:** Otolaryngology Head and Neck Surgery, University Hospitals Case Medical Center, Case Western Reserve University, Cleveland, Ohio, United States of America; University of Washington, Institute for Stem Cells and Regenerative Medicine, United States of America

## Abstract

Sildenafil citrate (Viagra), a phosphodiesterase 5 inhibitor (PDE5i), is a commonly prescribed drug for erectile dysfunction. Since the introduction of Viagra in 1997, several case reports have linked Viagra to sudden sensorineural hearing loss. However, these studies are not well controlled for confounding factors, such as age and noise-induced hearing loss and none of these reports are based on prospective double-blind studies. Further, animal studies report contradictory data. For example, one study (2008) reported hearing loss in rats after long-term and high-dose exposure to sildenafil citrate. The other study (2012) showed vardenafil, another formulation of PDE5i, to be protective against noise-induced hearing loss in mice and rats. Whether or not clinically relevant doses of sildenafil citrate cause hearing loss in normal subjects (animals or humans) is controversial. One possibility is that PDE5i exacerbates age-related susceptibility to hearing loss in adults. Therefore, we tested sildenafil citrate in C57BL/6J, a strain of mice that displays increased susceptibility to age-related hearing loss, and compared the results to those obtained from the FVB/N, a strain of mice with no predisposition to hearing loss. Six-week-old mice were injected with the maximum tolerated dose of sildenafil citrate (10 mg/kg/day) or saline for 30 days. Auditory brainstem responses (ABRs) were recorded pre- and post injection time points to assess hearing loss. Entry of sildenafil citrate in the mouse cochlea was confirmed by qRT-PCR analysis of a downstream target of the cGMP-PKG cascade. ABR data indicated no statistically significant difference in hearing between treated and untreated mice in both backgrounds. Results show that the maximum tolerated dose of sildenafil citrate administered daily for 4 weeks does not affect hearing in the mouse. Our study gives no indication that Viagra will negatively impact hearing and it emphasizes the need to revisit the issue of Viagra related ototoxicity in humans.

## Introduction

Sildenafil citrate (Viagra), a phosphodiesterase 5 inhibitor (PDE5i), was originally synthesized for angina pectoris. It was patented in 1996, approved by the FDA in 1998, and then prescribed for erectile dysfunction and pulmonary hypertension. Within the first month of release, 300,000 prescriptions were written and more than 400 million dollars worth sold within its first quarter in the US [1]; as of 2007, it was reported that 40 million prescriptions have been filled worldwide [2]; in 2010–2012, Viagra delivered at least $6 billion in revenues for Pfizer [3]. Now introduced in 50 countries with multiple PDE5i formulations (tadalafil or Cialis, vardenafil or Levitra), patents expiring globally, and investigations for off-label use, such as in cardiovascular disease [4] or altitude sickness [5], [6], we may not only see an increase in the amount of PDE5i prescribed but also more long-term side effects surfacing as a result.

One specific side effect that requires further investigation is sudden sensorineural hearing loss (SSHL). SSHL is characterized by a sudden or rapid loss of hearing, often causing distress in affected patients. The incidence of SSHL is approximately 4,000 cases per year with an unknown etiology but can be attributed to multiple underlying causes, such as viral infections or ototoxic drugs [7].

In 2007, Mukherjee *et al* presented a case report of SSHL, verified by audiometric data, in a 44-year-old man after he ingested sildenafil citrate at 50 mg/day for 15 days for erectile dysfunction [8]. As a response, the FDA evaluated its post-marketing data, indicating that 29 patients who presented with SSHL showed a temporal relationship with ingestion of sildenafil citrate. Of the 29 patients present, 12 on follow-up were reported to have ongoing hearing loss [9]. This prompted the FDA to revise Viagra’s safety label to include sudden hearing loss as a possible side effect [10]. Since then, subsequent case studies and retrospective chart reviews have surfaced suggesting that various formulations of PDE5i may induce SSHL [11], [12], [13]. However, none of the reports are based on a prospective double-blind clinical study. In 2009, Okuyucu *et al* enrolled 18 patients with erectile dysfunction who had been using a PDE5i in a prospective clinical study [14]. The study performed audiometric testing on all their patients before and 1, 5, and 72 hours after drug ingestion. They found significant hearing threshold increases, consistent with ototoxicity criteria, in 4 (22%) of their patients. Specifically, their patients had unilateral and reversible hearing loss 24 hours after ingestion. The time correlation between ingestion and hearing loss was consistent with retrospective studies done by Maddox *et al* and Khan *et al* in which they found 88% (N = 17) and 66.7% (N = 18) of their patients, respectively, to have hearing loss in the same time frame. However, it is unclear whether baseline audiometric data was taken into account for each of the reported cases in Maddox *et al* and Khan *et al* [11], [13]. Khan *et al* also indicates that 4 of the cases reported by Maddox *et al* were in fact previously diagnosed with otologic disease, indicating that the reported data might be confounded by other factors such as pre-existing co-morbidities. In particular, the target population (older adults) for PDE5i prescriptions are at an increased risk for age-related hearing loss (presbycusis). Therefore, more studies are necessary to determine the relationship between PDE5i and SSHL.

Rodents have served as excellent models in hearing research but three separate studies have found conflicting findings in the mouse or rat model with regard to PDE5i and hearing loss. Jaumann *et al* showed that in both a mice and rat model vardenafil protects against noise-induced hearing loss (NIHL) [15]. They demonstrated that treatment with a PDE5i reduced ABR threshold shifts observed in untreated mice and rats following acoustic trauma (4–16 kHz two octave band noise stimulus 120 dB SPL for 1 hour). Rats treated 2 hours prior to acoustic trauma or 6 hours after acoustic trauma with 10 mg/kg (MPK) or 0.1 to 3 MPK, respectively, showed significant resistance to NIHL. Similar results were found in mice treated at 10 MPK one hour prior to acoustic trauma. In addition, this study demonstrated *in vivo* that vardenafil activated the cGMP-Prkg1 signaling cascade in outer hair cells, satellite cells, and spiral ganglion nuclei, shielding the sensory epithelia in the cochlea from damage through the function of poly-ADP ribose polymerase (PARP). As a negative control, chronic administration of vardenafil (21 days) did not affect hearing function in rats as verified by audiometric testing. The data from Jaumann et al [15] suggest that PDE5 inhibitors may provide a new therapeutic option for NIHL in humans. In contrast, Hong *et al* showed that a daily dose of sildenafil citrate in rats at 10 MPK for 135 days increased 4 kHz tone bursts (TB) thresholds 15 days into the treatment, 8 kHz TB thresholds at 135 days, and click thresholds as early as 100 days [16]. Furthermore, this study found increased auditory middle latency responses (AMLRs) at 105 days in their sildenafil citrate treated rats compared to control. In support, Bakir *et al* recently reported elevated caspase 3 immunoreactivity in 30% of the sildenafil citrate treated rats, suggesting apoptotic activation from 45 days of sildenafil citrate treatment at 1.5 MPK [17]. However, this study failed to present physiological threshold changes to support its histological findings.

In light of these contradictory results, and with the ubiquitous use of sildenafil, this study attempts to clarify hearing function in chronic administration of sildenafil, which we define as a daily dose of sildenafil injected IP for 30 days.

## Materials and Methods

### Mice

Mice from two inbred genetic backgrounds were used in this study: C57BL/6J, which is genetically predisposed to age-related hearing loss (a model for presbycusis), and FVB/N, which is not predisposed to acquire hearing loss and considered to be a ‘good hearing’ strain (www.jax.org). For physiological data: The FVB/N mice were male and female (N = 4 treated with sildenafil and N = 4 treated with vehicle) at 6 weeks of age. The C57BL/6J mice were female and male (N = 7 treated with sildenafil and N = 9 treated with vehicle) at 6 weeks of age. For qRT-PCR analysis: 4-week-old C57BL/6J (N = 3 treated with sildenafil and N = 3 treated with vehicle) were both male and female. Ear punch was used to identify the animals.

### Ethics Statement

This study was carried out in strict accordance with the recommendations in the Guide for the Care and Use of Laboratory Animals of the National Institutes of Health and animal welfare guidelines at Case Western Reserve University (CWRU), USA. The protocol was approved by the Institutional Animal Care and Use Committee at CWRU (Protocol Number: 2012-0068).

### Drug Application

For the hearing experiments, we administered sildenafil citrate (Sigma Aldrich) dissolved in 0.9% NaCl solution at 2 mg/ml stored at 4° degrees Celsius for up to one week. This solution was administered daily intraperitoneal (I.P.) at 10 mg per kg of body weight (MPK) for 30 days. 10 MPK was chosen since it was a previously studied concentration [15], [16]. Furthermore, 10 MPK is considered the maximum tolerated dose, approximately 0.6 times the maximum recommended human dose of 100 mg on an mg/m^2^ basis [18]. 0.9% NaCl solution was used as a vehicle at equal volumes. For qRT-PCR experiments, we administered 10 MPK sildenafil citrate dissolved in 0.9% NaCl solution or vehicle at equal volumes once twenty-four hours prior to sacrifice.

### Hearing Measurements

ABR recordings were conducted as previously described [19]. Briefly, mice were anesthetized with dilute ketamine, xylazine, and acepromazine solution (15 mg ketamine, 3 mg xylazine, 0.5 mg acepromazine and sterile water/saline) I.P. alternating side of ejection if possible. Then platinum subdermal needle electrodes were inserted ventrolaterally to the right and left ear. To assess cochlear function, hearing was tested at frequencies representing the low (8 kHz), middle (16 kHz) and high (32 kHz) frequency regions of the mouse cochlea. Pure tone pips at 8, 16 or 32 kHz was presented to the external ear of the mouse starting at 100-decibel sound pressure level (dB SPL) and decreasing by 10 dB steps or 5 dB steps; this sequence was repeated until the lowest intensity that evoked a reproducible ABR waveform was detected. The stimulus was presented for 100 ms duration and for 750 sweeps to both the left and right ear (one at a time) through high-frequency transducers. For each mouse, ABR thresholds were averaged across left and right ear for statistical analysis.

### qRT-PCR

Mice were euthanized and the cochlea were dissected and immediately stored at −80°C [20]. Total RNA was isolated by Trizol (Invitrogen, Carlsbad, CA, USA) according to the manufacturer’s protocol. The extracted RNA was quantitated with NanoDrop spectrophometer and RNA quality was analyzed using Agilent Bioanalyzer with RNA Nano Chip 6000. Reverse transcription was performed with SA Biosciences (Frederick, MD, USA) RT2 First Strand Kit (C-03), which contains an effective genomic DNA elimination step and built-in external RNA control. RNA input was 700 ng per RT reaction. qRT-PCR was performed on the cDNA templates generated in an iCycler Bio-Rad in 96 well format in a volume of 24 µl per reaction. SYBR green dye was used for qRT-PCR. PARP primers were used (5′-AGAGAAGCCACAGCTGGGTA-3′ and 5′-CATCCACCTCGTCACCTTTT-3′), and GAPDH (5′-ATGTGTCCGTCGTGGATCTGAC-3′ and 5′-AGACAACCTGGTCCTCAGTGTAG-3′). The amount of cDNA template in each reaction was 10 ng of input RNA. Assays were performed in triplicate and data analysis was performed via Excel. The ΔΔCt method was employed to calculate fold changes. ΔCt represents normalized gene expression level (normalized with an internal control housekeeping gene GAPDH). ΔCt for vehicle and sildenafil samples were averaged and then used to calculate ΔΔCt. CV, or coefficient of variation, is calculated using the formula STDEV/average.

## Results

In the laboratory mouse, hearing reaches adult levels of sensitivity around thirty days after birth and ABR threshold for normal (untreated) mice is typically 30±5 dB SPL [21], [22]. A 10 dB SPL increase in ABR threshold would mean a ten-fold increase in stimulus intensity necessary to elicit an auditory-evoked brainstem response or ten-fold decrease in hearing sensitivity. A drug could be considered potentially ototoxic if it induces a threshold shift of ≥10 dB SPL. Hearing status of each mouse was verified prior to first injection (considered baseline or ‘day 0’ data). To evaluate cochlear function in mice after sildenafil treatement, hearing was tested at frequencies representing the high (32 kHz), middle (16 kHz) and low (8 kHz) frequency regions of the mouse cochlea. The reasons for this approach are as follows. Sampling three different regions along the tonotopic gradient provides better evaluation of the cochlear function in mice and recording ABRs at 8, 16 and 32 kHz is a standard practice in mice [23], [24]. Also, reports show that ototoxic damage in humans, with drugs such as cisplatin, usually occurs first near the basal turn of the cochlea or the high-frequency region and progresses to lower frequencies over time [25], and studies in humans have used primary outcome measure based on ABR recordings at frequencies representing the high (8 kHz), middle (4 kHz) and low (2 kHz) frequency regions of the human cochlea [26].

**Figure 1:**
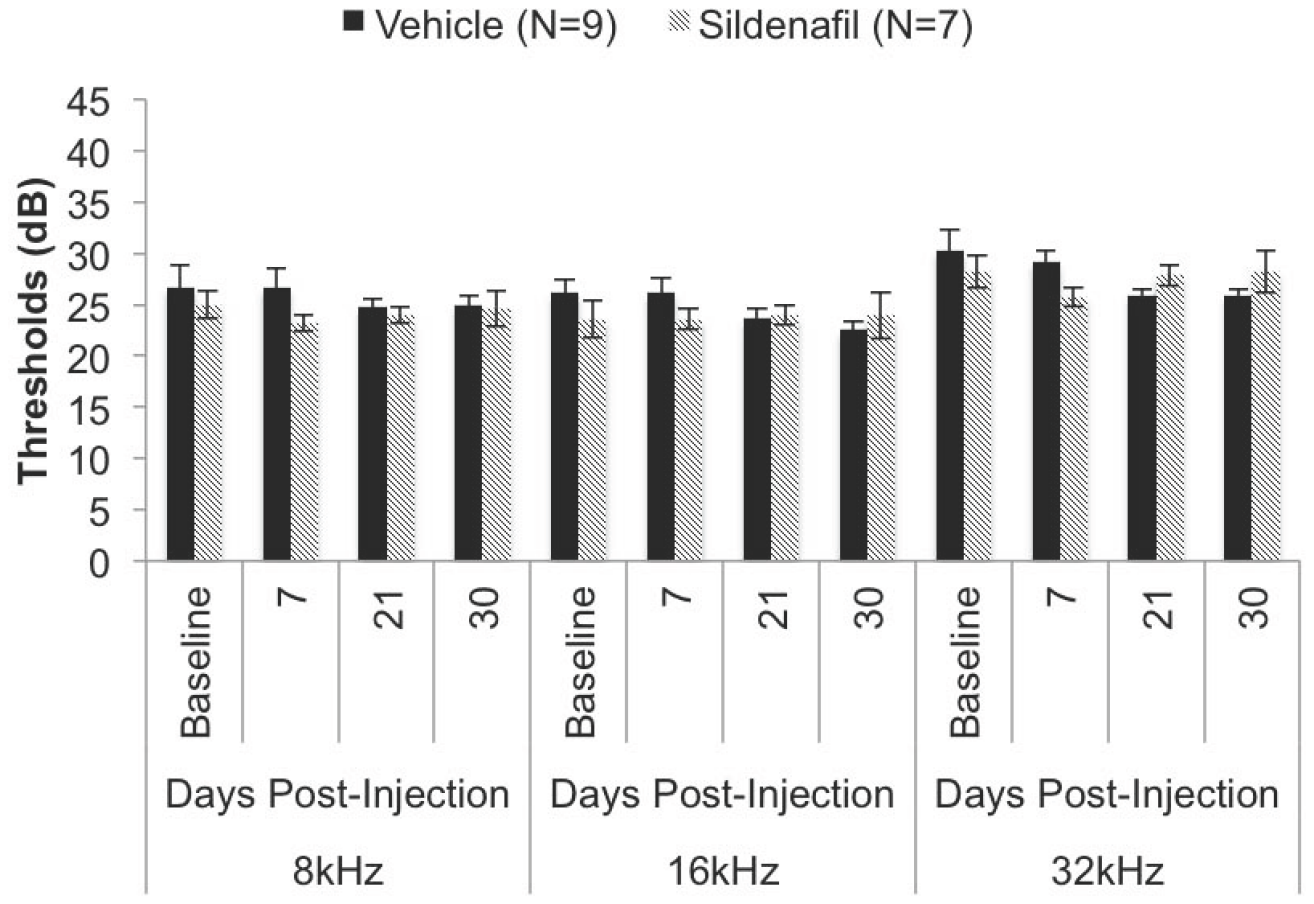
Hearing thresholds in the C57BL/6J mice after treatment with sildenafil. Hearing thresholds were determined across three frequencies (8, 16, 32 kHz) and on day 0 (baseline), 7, 21 and 30. Table 1 indicates the relative *P* values for each time point with corresponding hearing thresholds. Vehicle (N = 9) and sildenafil citrate (N = 7) C57BL/6J mice were 1.5 months of age at initial injection at 10 MPK.

**Figure 2:**
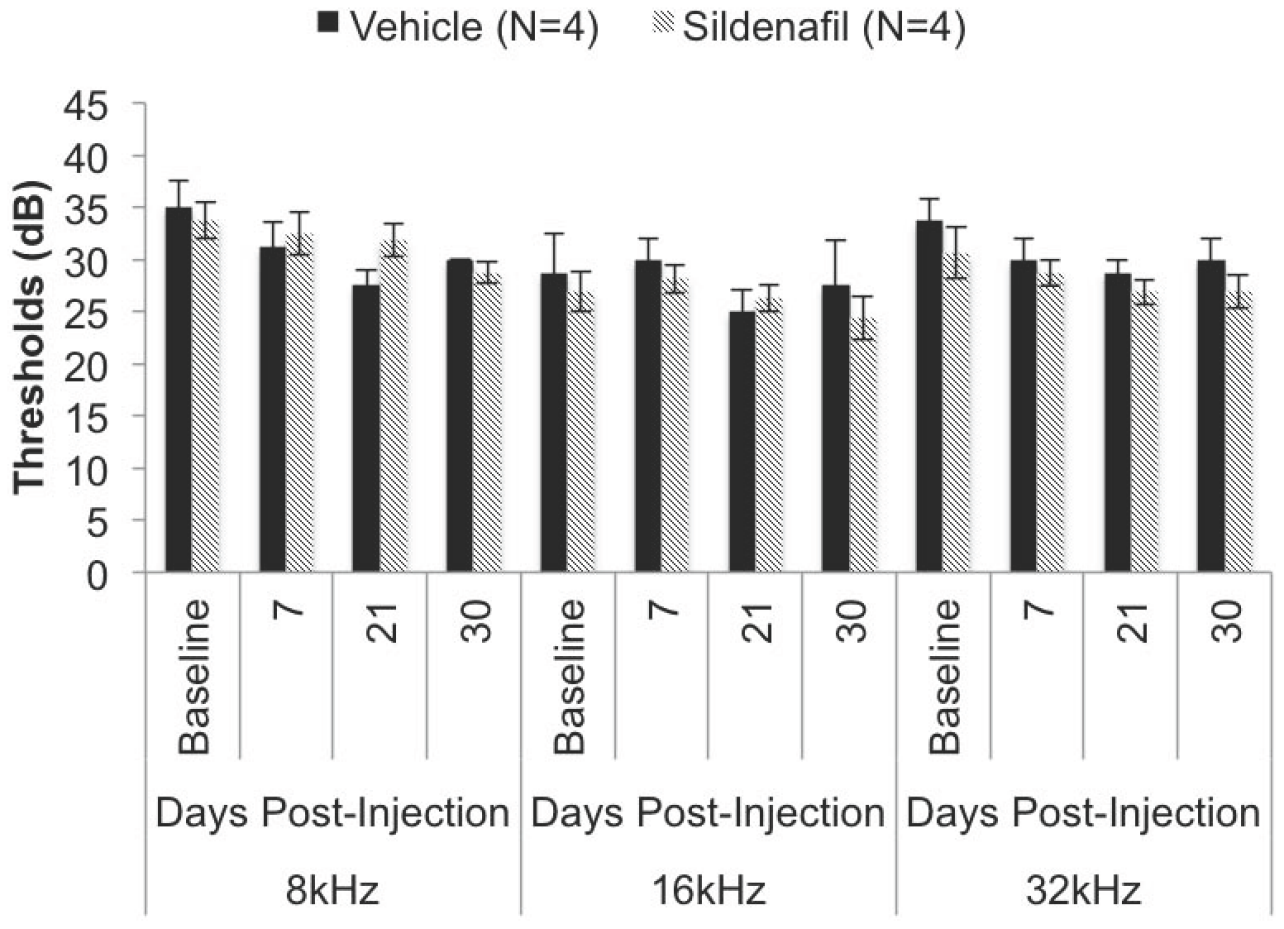
Hearing thresholds in the FVB/N mice after treatment with sildenafil. Hearing thresholds were determined across three frequencies (8, 16, 32 kHz) and on day 0 (baseline), 7, 21 and 30. Table 2 indicates the relative *P* values for each time point with corresponding hearing thresholds. Vehicle (N = 4) and sildenafil citrate (N = 4) FVB/N mice were 1.5 months of age at initial injection at 10 MPK.

**Figure 3:**
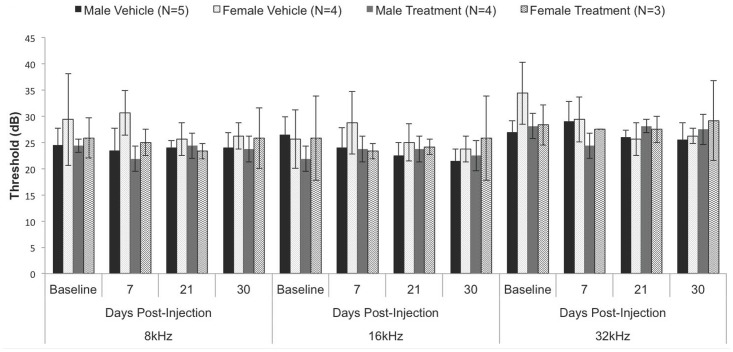
Hearing thresholds in males versus female in the C57BL/6J mice after treatment with sildenafil. C57BL/6J data (shown in Figure 1) separated by gender to demonstrate no significant difference.

We carried out longitudinal evaluation of hearing during treatment: Our results indicate that, within the mouse model, chronic, systemic (IP) administration (30 days) of sildenafil citrate at a concentration of 10 MPK does not affect hearing sensitivity in C57BL/6J (Fig. 1) or FVB/N (Fig. 2). Our study shows no significant changes in ABR thresholds between vehicle and sildenafil citrate treated mice in either C57BL/6J or FVB/N strain (Table 1 & 2). To discern whether female mice have estrogen-mediated protection, we separated the C57BL/6J data by sex with no statistically significant difference between males and females in their respective vehicle or treatment group (Figure 3). Similar results were observed with males and females in the FVB/N mice. However, we do not have sufficient number of males and females in the FVB/N group to do a statistical analysis (data not shown).

**Table 1:** Hearing in C57BL/6J mice treated with sildenafil citrate.

Frequency	Days post- Treatment	Hearing Thresholds (dB SPL) ofmice treated with vehicle (N = 9)	Hearing Thresholds (dB SPL) of micetreated with sildenafil citrate (N = 7)	*P* value*
8 kHz	0	26.67±9.07	25.00±5.19	0.09
	7	26.67±7.86	23.21±3.16	
	21	24.72±3.63	23.93±2.89	
	30	25.00±3.83	24.64±6.64	
16 kHz	0	26.11±5.57	23.57±6.91	0.2
	7	26.11±6.31	23.57±3.63	
	21	23.61±4.47	23.93±3.49	
	30	22.50±3.54	23.93±8.36	
32 kHz	0	30.28±8.48	28.21±6.08	0.35
	7	29.17±6.31	25.71±3.31	
	21	25.83±4.47	27.86±3.78	
	30	25.83±3.54	28.21±7.75	

C57BL/6J mice at 45 days after birth were treated with vehicle (N = 9) or sildenafil (N = 7). Hearing threshold value in each cell in column 3 represents average ABR threshold derived from 9 mice (N = 9) at that time point. ABR thresholds were recorded from the same group of mice over time, day 0, 7, 21 and 30 (also referred to as ‘longitudinal’ testing), to assess cumulative effects of sildenafil treatment on hearing. It should be noted that ‘day 0’ is pre-treatment or baseline ABR. The same explanation holds for column 4 (treated mice, N = 7). *A repeated measure ANOVA, non parametric, using a Friedman test was used to compare the hearing thresholds of the mice over time. A *P* value less than 0.05 is considered significant.

**Table 2:** Hearing in FVB/N mice treated with sildenafil citrate.

Frequency	Days post- Treatment	Hearing Thresholds (dB SPL) ofmice treated with vehicle (N = 4)	Hearing Thresholds (dB SPL) of micetreated with sildenafil citrate (N = 4)	*P* value
8 kHz	0	35±5.00	33.75±5.18	0.8
	7	31.25±2.89	32.5±6.55	
	21	27.5±2.89	31.88±4.58	
	30	30±0	28.75±3.54	
16 kHz	0	28.75±7.5	26.88±4.58	0.5
	7	30±4.08	28.13±3.72	
	21	25±4.08	26.25±3.54	
	30	27.5±8.66	24.38±4.17	
32 kHz	0	33.75±4.08	30.63±7.76	0.3
	7	30±4.08	28.75±3.54	
	21	28.75±2.5	26.88±3.72	
	30	30±4.08	26.88±4.58	

FVB/N mice at 45 days after birth were treated with vehicle (N = 4) or sildenafil (N = 4). Hearing thresholds were compared and *P* values were determined as described in the legend to Table 1).

### Determining Activation of Sildenafil Target in Mouse Cochlea

The PDE5i mechanism of action has been well described in the literature [27], [28]. Nitric oxide (NO) activates guanylate cyclase, allowing the conversion of guanosine triphosphate (GTP) into cyclic guanosine monophosphate (cGMP). To regulate this cycle, phosphodiesterase cleaves cGMP into guanosine monophosphate (GMP). As the name implies, phosphodiesterase 5 inhibitors selectively inhibit one isoform of the regulatory phosphodiesterases [27]. cGMP then acts as a second messenger to activate cGMP-dependent protein kinase (PKG), which regulates the contractile activity of smooth muscle cells by controlling intracellular calcium concentrations [28]. In the rodent cochlea, it has been shown that PDE5 inhibitor vardenafil increases levels of poly ADP-ribose polymerase (PARP), a downstream marker of cGMP-PKG cascade [15].

**Figure 4:**
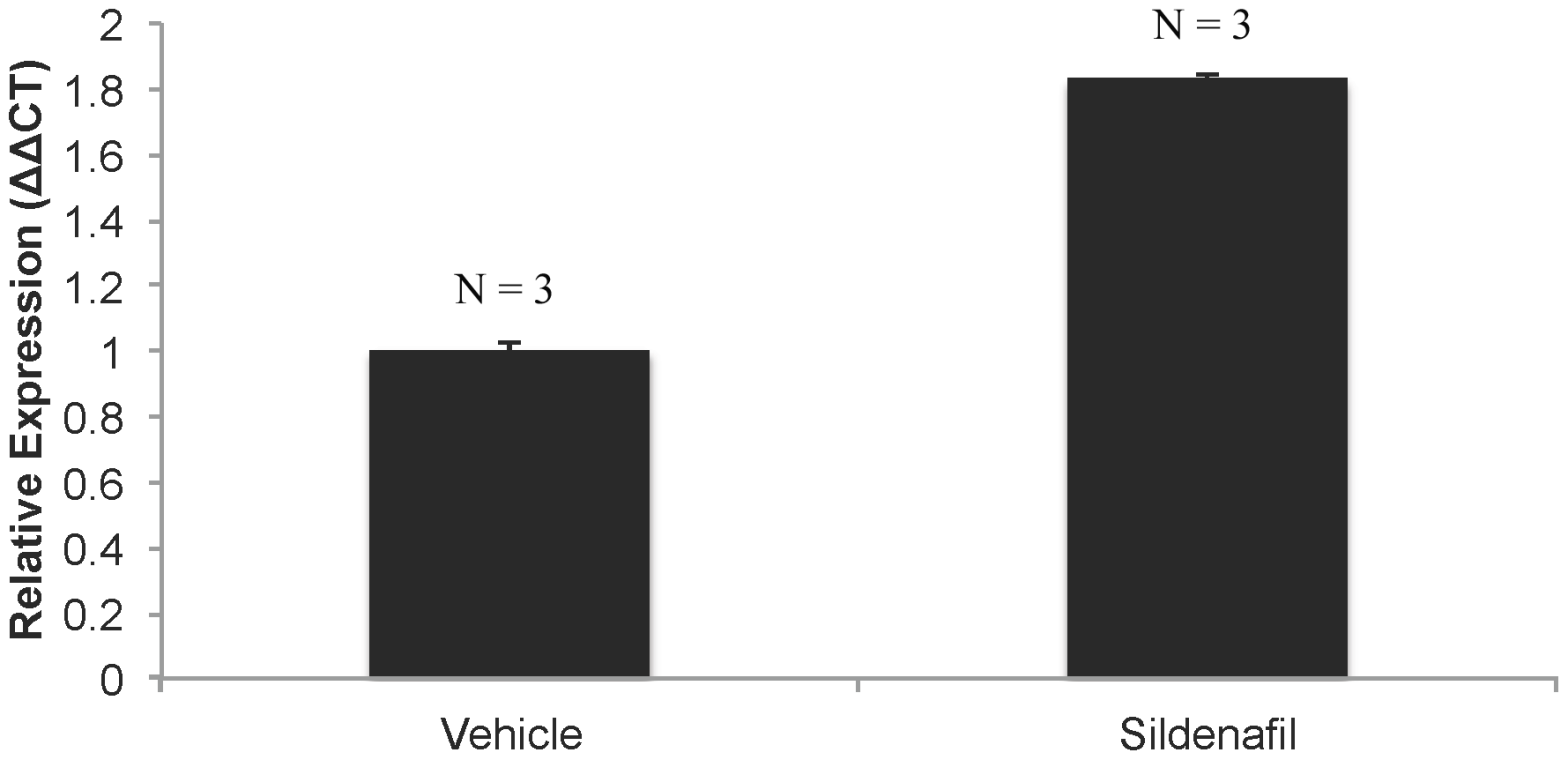
PARP mRNA level in the mouse cochlea following sildenafil injection. One-month-old C57BL/6J mice were injected I.P. at 10 MPK (vehicle N = 3 and sildenafil citrate N = 3). They were sacrificed 24 hours after sildenafil injection and qRT-PCR was performed on RNA isolated from cochlear tissue. The relative expression of PARP mRNA is presented. A fold change equals 2^(-ΔΔCt)^.

Inability for activated sildenafil citrate to enter the cochlea could explain lack of hearing loss in the treated groups. In order to verify whether administered sildenafil citrate crossed the blood-cochlear barrier in an active form, we performed a quantitative real-time RT-PCR to quantify the level of PARP mRNA as a marker for penetrance and activity of sildenafil citrate. Twenty-four hours after an injection with sildenafil citrate at 10 MPK or equivalent vehicle into wild-type C57BL/6J mice, we found a 1.8 fold increase in PARP mRNA expression in the cochlea following sildenafil treatment (Figure 4). The co-efficient of variation was 0.00–0.02, suggesting negligible variation between replicates and reliable results that are biologically significant.

## Discussion

Our study found that the chronic administration of PDE5i was not ototoxic at any time point for up to 30 days in the mouse model. One novel approach that our study uses is treating and testing two genetic backgrounds of mice. To test the possibility that PDE5i associated hearing loss is linked to age-related susceptibility to hearing loss we tested sildenafil citrate in C57BL/6J, a strain of mice that displays increased susceptibility to age-related and noise-induced hearing loss [29], and compared the results to those obtained from the FVB/N, a strain of mice with no reported predisposition to hearing loss. However, no significant difference in hearing thresholds was seen at baseline, 7 days, 21 days, and 30 days in the C57BL/6J line compared to the untreated C57BL/6J mice or the treated FVB/N strain. Since both male and female mice were used in this study the potential influence of estrogen on cGMP mediated otoprotection had to be considered. However, evidence presented here (Fig. 3) did not support estrogen-mediated otoprotection. Therefore, even in a susceptible background, sildenafil citrate did not induce hearing loss.

Jaumann *et al* reported that vardenafil protects against noise-induced hearing loss (NIHL) in rodent models. Jaumann *et al* show that one of the key downstream mechanisms of protection against noise-induced hearing loss (NIHL) is PARP activation via the cGMP-PKG [15] . The study demonstrated the presence of PDE5 and Prkg1a and Prkg1b within the cochlear hair cells, spiral ganglion neurons, and satellite cells in the rats and mice. In their Prkg1 knockout mice, one hour of acoustic trauma caused a significant increase in hearing thresholds, suggesting that Prkg1 is an important mediator in hearing loss due to NIHL. The study found that vardenafil treated mice and rats showed significant resistance to NIHL compared to untreated mice and saw an increase in PARP IHC stained cells in the vardenafil treated mice and rats [15]. Findings from our study and that of Jaumann *et al* suggest revisiting PDE5i related otoxoticity in humans.

Two important factors that need further delineation are: 1) the temporal relationship between PDE5i ingestion and onset of symptoms and 2) dosing. The clinical studies previously described determined that PDE5i consumption caused SSHL based on the presentation of symptoms relative to the time of drug ingestion. Specifically, they found that the majority of the SSHL diagnosed was approximately 24 hours after ingestion. This suggests that the mechanism of ototoxicity is rapid in onset with an acute sequelae. Therefore, we would expect a rather acute process to present similarly in the mouse model. However, this is counter to the Hong *et al* findings; their study found statistically significant findings at 105 and 135 days of chronic 10 MPK injections in rats, with the earliest changes in hearing occurring fifteen days after the initial injection [16]. The ABRs performed 5 days after initial injection showed no statistically significant changes, suggesting sub-acute ototoxicity. One possible explanation is that over time, the drug or its metabolites accumulated, causing an indirect ototoxicity, which could theoretically occur with any drug.

Separately, the clinical reports do not report PDE5i dosing regimens. Dosing of sildenafil citrate for erectile dysfunction (ED) usually begins at 50 mg once, while dosing for pulmonary hypertension (PAH) usually starts at 20 mg three times a day [30]. Therefore, the current studied dosing of 10 MPK in mice or 60 mg on an mg/m^2^ basis in humans [18] is greater than the current standard of care. Future studies are necessary to understand how the amount of sildenafil citrate or PDE5i utilized correlates to the degree of hearing loss.

## Conclusion

Our study shows that chronic administration (4 weeks) of sildenafil citrate is not sufficient to cause hearing loss in the mouse model. The results presented here gives no indication that sildenafil citrate will affect hearing in humans. However, to ultimately determine whether PDE5i induces SSHL in a human population, a prospective double-blind clinical study would need to be performed that thoroughly investigates the dosing and temporal relationship of a PDE5i and controls for age-and noise induced hearing loss in the adult population.
